# AMP-IBP5: A Multifunctional Antimicrobial Peptide for Advanced Wound Healing and Inflammatory Skin Disorders

**DOI:** 10.3390/jfb16050174

**Published:** 2025-05-12

**Authors:** Alafate Abudouwanli, Ge Peng, Mengyao Yang, Wanchen Zhao, Quan Sun, Shan Wang, Yi Tan, Arisa Ikeda, Hideoki Ogawa, Ko Okumura, François Niyonsaba

**Affiliations:** 1 Atopy (Allergy) Research Center, Juntendo University Graduate School of Medicine, Tokyo 113-8421, Japan; a.alafate.vg@juntendo.ac.jp (A.A.);; 2 Department of Dermatology, The First Hospital of China Medical University, Shenyang 110001, China; 3 Department of Dermatology, Beijing Children’s Hospital, Capital Medical University, National Center for Children’s Health, Beijing 100045, China; 4 Department of Nephrology, Juntendo University Graduate School of Medicine, Tokyo 113-8421, Japan; 5 Faculty of International Liberal Arts, Juntendo University, Tokyo 113-8421, Japan

**Keywords:** AMP-IBP5, antimicrobial peptide, wound healing, atopic dermatitis, psoriasis, inflammatory pathway

## Abstract

Wound healing is a complex, multiphase process crucial for restoring tissue integrity and functionality after injury. Among the emerging therapeutic approaches, antimicrobial peptides (AMPs) have shown substantial promise because of their dual role in microbial defense and cellular modulation. AMP-IBP5, a novel AMP derived from insulin-like growth factor-binding protein 5, exhibits both antimicrobial and wound-healing properties, making it a promising therapeutic candidate. This peptide exhibits robust antimicrobial activity, augments keratinocyte proliferation, increases fibroblast migration, induces angiogenesis, and modulates the immune response. Mechanistically, AMP-IBP5 activates Mas-related G protein-coupled receptors and low-density lipoprotein receptor-related protein 1 (LRP1) in keratinocytes, stimulating IL-8 production and vascular endothelial growth factor expression to accelerate wound healing. This molecule also interacts with LRP1 in fibroblasts to increase cell migration and promote angiogenesis while mitigating inflammatory responses through targeted cytokine modulation. Preclinical studies have demonstrated its remarkable efficacy in promoting tissue repair in diabetic wounds and inflammatory skin conditions, including atopic dermatitis and psoriasis. This review delves into the broad therapeutic potential of AMP-IBP5 across dermatological applications, focusing on its intricate mechanisms of action, comparative advantages, and its path toward clinical and commercial application.

## 1. Introduction

The skin serves as a fundamental barrier, safeguarding the body against environmental insults and maintaining homeostasis. Skin integrity is often compromised by trauma, burns, infections, skin diseases, and metabolic dysfunction, necessitating a tightly regulated wound healing process that comprises the phases of hemostasis, inflammation, proliferation, and remodeling [1,2]. Classically, cutaneous wound healing begins with an inflammatory response to injury [3]. This initial inflammatory phase clears pathogens, paving the way for subsequent tissue regeneration [4]. During the regenerative phase, key processes such as epithelialization, angiogenesis, and the deposition of collagen culminate in a remodeling process to restore the tissue [5]. However, failure of these mechanisms underlying tissue recovery can lead to chronic, nonhealing wounds, such as diabetic foot ulcers, pressure ulcers, and venous leg ulcers. To date, approximately 1–2% of the global population suffers from chronic wounds, which present immense physical, emotional, and financial burdens, posing major challenges to healthcare systems worldwide [6], with economic burdens exceeding USD 25 billion annually in the U.S. alone [7]. In England, leg ulcers incur a staggering GBP 3.1 billion annually in healthcare costs [8]. Given the prevalence and impact of chronic wounds, effective therapeutic interventions are critically needed to optimize wound-healing outcomes [9].

Since the beginning of this century, antimicrobial peptides (AMPs) have garnered substantial interest due to their multifaceted biological roles [10]. In the wound-healing process, in addition to their direct antimicrobial effects, AMPs influence diverse cellular pathways, including cytokine signaling, angiogenesis, and re-epithelialization. In recent years, AMP-IBP5, derived from insulin-like growth factor-binding protein 5 (IGFBP-5), has emerged as a promising therapeutic agent in dermatology, functioning as both an antimicrobial agent and a modulator of wound healing [11,12]. This review synthesizes current knowledge regarding AMP-IBP5 and its prospective role in advancing dermatological care.

## 2. Overview of Skin-Derived AMPs in Wound Healing

AMPs are a diverse class of polypeptides that are typically composed of fewer than 50 amino acid residues [13]. As of early 2025, more than 3300 natural AMPs have been identified, reflecting their remarkable abundance across diverse organisms [14]. Key AMPs active in human skin include defensins, cathelicidin LL-37, dermcidin-1, dermcidin-1L, α-melanocyte stimulating hormone, and lactoferricin, which play a critical role in both innate and adaptive immune responses [15,16,17]. These peptides are predominantly produced by keratinocytes, neutrophils, and sebocytes, and their expression is markedly upregulated in response to injury or infection. In such contexts, they not only provide immediate antimicrobial defense but also orchestrate cellular responses that facilitate wound repair [18].

AMPs typically possess a net positive charge and exhibit an amphiphilic structure, which enables strong interactions with negatively charged surfaces and membranes, thereby facilitating membrane insertion, pore formation, and the subsequent leakage of intracellular components [13,19,20]. This distinctive structural property confers broad-spectrum antimicrobial activity of AMPs, enabling them to effectively target various pathogenic organisms, including bacteria, fungi, and viruses [21,22]. In addition to membrane disruption, AMPs also exert antimicrobial effects by interacting with intracellular targets, such as inhibiting the synthesis of critical cellular components, including cell walls, nucleic acids, and proteins [23]. For example, human β-defensin (hBD)-3 mediates a bactericidal effect against *Staphylococci* in part by impairing cell wall biosynthesis [24], while LL-37 can exert an antibacterial effect through a distinct mechanism, such as binding to bacterial DNA and interfering with its replication and transcription processes [25]. Furthermore, histatin 5 causes the cell death of *Candida albicans* by triggering mitochondrial dysfunction and impairing ATP synthesis, leading to cellular bioenergetic collapse [26]. In addition to their direct antimicrobial action, AMPs are known to have a wide range of immunomodulatory functions, contributing to host defense by shaping both innate and adaptive immune responses [27,28]. Specifically, AMPs influence host immune responses by inducing cell migration, proliferation, and differentiation [29,30,31,32]. Furthermore, AMPs modulate cytokine and chemokine production, promote angiogenesis and wound healing, and maintain skin barrier function as well as a stable and beneficial microbial balance in the skin [32,33,34,35,36,37,38,39].

**Figure 1:**
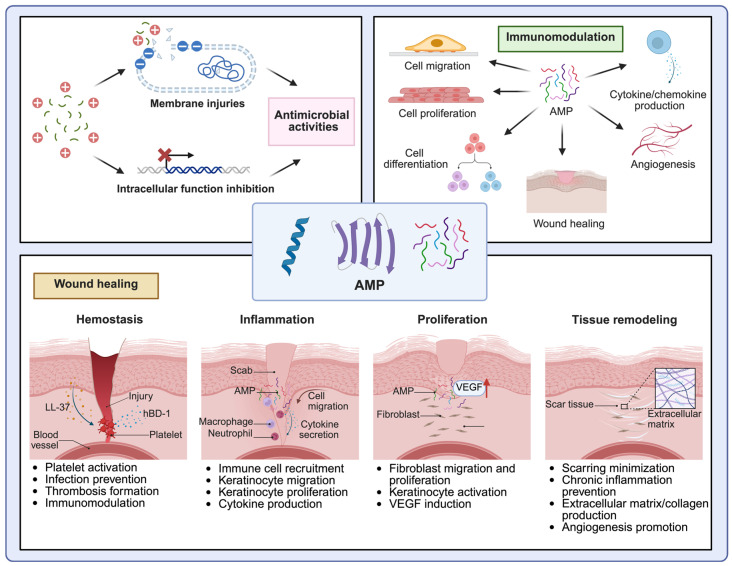
Multiple biological functions of skin-derived AMPs during the wound-healing process. Skin-derived AMPs play multifaceted roles in wound healing by exerting antimicrobial activities and modulating host immune responses. AMPs disrupt microbial membranes through electrostatic interactions or penetrate microbial cells to interfere with intracellular targets such as nucleic acids and protein synthesis. In addition to their antimicrobial effects, AMPs promote host immunity by promoting cell migration, proliferation, and differentiation; cytokine and chemokine production; angiogenesis; and overall wound healing. AMPs stimulate wound healing by involving all four stages of the wound-healing process. During the hemostasis phase, LL-37 promotes platelet activation and thrombus formation, contributing to rapid bleeding control. Platelets express hBD-1 in response to bacterial stimuli to provide early antimicrobial protection. In the inflammation stage, AMPs such as hBD-2 and hBD-3 recruit immune cells, stimulate cytokine production, and enhance keratinocyte motility, thereby promoting re-epithelialization and accelerating wound closure. During proliferation, LL-37 facilitates angiogenesis by upregulating VEGF, whereas hBD-3 supports fibroblast migration and keratinocyte proliferation. In the remodeling phase, AMPs facilitate the resolution of inflammation and support matrix remodeling by regulating cytokine production, minimizing scarring, and promoting the synthesis and reorganization of collagen and extracellular matrix components, ultimately contributing to the restoration of the epidermal barrier and functional recovery. Created with BioRender.com “https://BioRender.com/ (accessed on 19 February 2025)”.

Wound healing is a highly coordinated biological process generally comprising four key stages: hemostasis, inflammation, proliferation, and tissue remodeling [40]. Each phase is characterized by distinct cellular and molecular activities that collectively aim to restore tissue integrity and functionality. During hemostasis, LL-37 has been shown to activate a range of platelet functions and promote thrombus formation, thereby contributing to effective bleeding control in a mouse model [41,42]. Activated platelets release a variety of bioactive molecules, including platelet-derived growth factor, transforming growth factor-β, and vascular endothelial growth factor (VEGF), to initiate and coordinate subsequent phases of the wound-healing process [43]. In addition, upon stimulation with agents such as *Staphylococcus aureus* α-toxin, platelets express hBD-1, which contributes to antimicrobial defense by inhibiting pathogen growth [44]. In the inflammation stage, AMPs play a pivotal role in recruiting immune cells, such as neutrophils and monocytes, to the wound site, facilitating the clearance of pathogens and necrotic debris [45,46]. For instance, hBD-2 enhances keratinocyte cytokine production and migration capacity, which, in turn, contributes to re-epithelialization during wound healing [29,47]. Topical administration of hBD-3 has been shown to accelerate wound closure by up to 40% in animal models, primarily through enhanced cytokine secretion, cell migration, and proliferation [32]. In the proliferation phase, angiogenesis and re-epithelialization are driven by fibroblast migration and keratinocyte activity, advancing the wound toward functional tissue repair [48,49,50,51]. LL-37, for example, upregulates VEGF expression by up to 60%, thereby facilitating angiogenesis in the context of wound healing [39]. hBD-3 further promotes fibroblast migration and keratinocyte proliferation to contribute to more efficient tissue regeneration. Skin-derived AMPs are equally critical during the remodeling phase, resolving inflammation and reestablishing epidermal barrier function. AMPs such as esculentin-1a(1-21)NH_2_ and SR-0379 may accelerate wound healing by increasing collagen deposition and production [33,52]. In addition, AMPs minimize scarring and prevent chronic inflammation by modulating cytokine production and balancing immune responses [1,32]. These characteristics underscore their therapeutic utility, with AMP-IBP5 emerging as a particularly compelling candidate (Figure 1).

## 3. AMP-IBP5 and Its Role in Wound Healing

AMP-IBP5 is derived from insulin-like growth factor-binding protein 5 (IGFBP-5), a member of the IGFBP family, through specific proteolytic processing mediated by two prohormone convertases (PCs), PC1/3 and PC2 [12]. The IGFBP family consists of six proteins (IGFBP-1 to IGFBP-6) that regulate a range of biological processes, such as cell proliferation, differentiation, adhesion, and migration, through both insulin-like growth factor (IGF)-dependent pathways and IGF-independent mechanisms [53]. In humans, IGFBP-5 is broadly expressed across multiple tissues, including the kidney, ovary, muscle, bone, lung, brain, and skin [54]. In the skin, IGFBP-5 is predominantly localized in keratinocytes and fibroblasts, implying a potential biological role of AMP-IBP5 in cutaneous physiology [55].

AMP-IBP5 is characterized by an amphipathic structure and a strong net positive charge of +7 at pH 7.0, features that are critical for its interaction with negatively charged microbial membranes [12]. These physicochemical properties endow AMP-IBP5 with the ability to selectively associate with bacterial membranes via electrostatic interaction while minimizing cytotoxicity toward host cells. This selective binding leads to membrane destabilization, increased permeability, and, ultimately, bacterial cell lysis. AMP-IBP5 exhibits broad-spectrum antimicrobial activity with efficacy comparable to that of LL-37 and superior to that of hBD-2 across multiple bacterial strains. Specifically, AMP-IBP5 demonstrated greater activity than both peptides against *Micrococcus luteus* and *Pichia pastoris* GS115, while against *S. aureus* 209P, *Escherichia coli* B, and *E. coli* kp, AMP-IBP5 activity was lower than that of LL-37 but exceeded that of hBD-2 [12]. In addition to its amphipathic nature and net positive charge, the C-terminal amide modification plays a critical role in enhancing the antimicrobial efficacy of AMP-IBP5. This structural feature has been widely recognized as a determinant of antimicrobial potency across various classes of AMPs [56]. A modified variant of AMP-IBP5 with a C-terminal glycine extension that lacks the amide group exhibited a complete loss of antimicrobial activity against *S. aureus* 209P, *E. coli* B, *E. coli* kp, *Enterococcus hirae*, or *S. saprophyticus* KD. Nevertheless, the efficacy of the AMP-IBP5 variant remained comparable to the intact peptide when tested against *M. luteus* and *P. pastoris* GS115. Notably, the antimicrobial activity of C-terminal-modified AMP-IBP5 toward *E. coli* K12 was reduced by approximately ten-fold [12]. These findings collectively underscore the critical role of the C-terminal amide group in preserving the full antimicrobial potency of AMP-IBP5, particularly against certain bacterial strains. In addition, the contribution of disulfide linkage to AMP structural integrity warrants consideration. For example, hBD-2 is a cysteine-rich AMP known to be stabilized by three intramolecular disulfide bonds [57]. As expected, disruption of the disulfide bonds in hBD-2 led to a marked reduction in its antimicrobial activity, with the carbamidomethylated form (CAM)-modified variant retaining only weak activity against *M. luteus* and exhibiting more than a five-fold reduction in potency compared to the intact form. In contrast, CAM-modified AMP-IBP5 showed a more modest reduction in antimicrobial activity. Although its efficacy against *S. aureus* 209P and *E. coli* kp was abolished, the peptide maintained comparable activity to the intact form against *M. luteus*, *E. coli* B, and *P. pastoris* GS115 and exhibited only a two-fold decrease in potency against *E. coli* K12 [12]. These findings suggest that the disulfide bond in AMP-IBP5 is less essential for maintaining antimicrobial function than in hBD-2, likely due to the presence of only a single disulfide linkage in the former. This structural resilience may contribute to the broader functional versatility of AMP-IBP5. Moreover, hemolysis assays revealed that AMP-IBP5 caused only 0.3% erythrocyte lysis at a concentration of 20 μM, markedly lower than the 4.1% lysis induced by LL-37 at 10 μM, underscoring AMP-IBP5’s superior biocompatibility [12].

**Figure 2:**
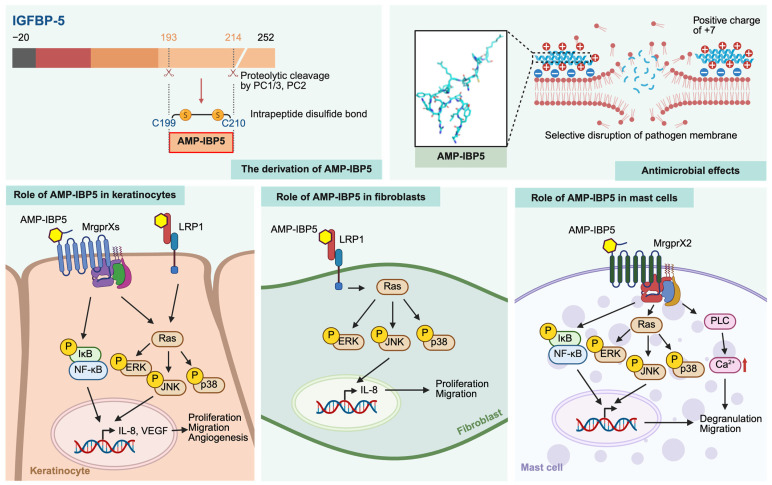
AMP-IBP5 and its role in wound healing. AMP-IBP5 is generated from IGFBP-5 through proteolytic cleavage by the prohormone convertases PC1/3 and PC2, resulting in an amphipathic peptide with a net positive charge at neutral pH and two cysteine residues that form an intramolecular disulfide bond. This unique structure of AMP-IBP5 enables selective interaction with negatively charged microbial membranes, conferring broad-spectrum antimicrobial activity while sparing host cells. Beyond its antimicrobial effects, AMP-IBP5 plays a pivotal role in wound repair by modulating various cellular and immunological processes. In keratinocytes, AMP-IBP5 promotes keratinocyte proliferation and migration through the activation of MrgprX2 and interaction with LRP1, which stimulates IL-8 production and VEGF expression, thereby enhancing re-epithelialization and angiogenesis. In fibroblasts, AMP-IBP5 engages with LRP1 to increase cellular migration and proliferation. Additionally, AMP-IBP5 modulates local immune responses by inducing mast cell degranulation and migration through MrgprX2 signaling, leading to the release of proangiogenic mediators that further contribute to tissue regeneration. Created with BioRender.com https://BioRender.com/ (accessed on 19 February 2025).

Beyond its antimicrobial efficacy, AMP-IBP5 modulates several key processes involved in wound healing, such as keratinocyte and fibroblast proliferation, migration, angiogenesis, and mast cell activation, demonstrating its multifaceted potential as a therapeutic agent [58,59,60]. Specifically, AMP-IBP5 promotes keratinocyte proliferation and migration by the activation of Mas-related G protein-coupled receptor X2 (MrgprX2) and low-density lipoprotein receptor-related protein 1 (LRP1), which, in turn, contribute to the healing process by inducing IL-8 production and upregulating VEGF expression [58]. This dual action not only accelerates cellular migration and proliferation but also facilitates angiogenesis, ensuring effective tissue repair. Through its interaction with LRP1, AMP-IBP5 also facilitates fibroblast activation, promoting both migratory and proliferative responses [59]. Additionally, AMP-IBP5-mediated activation of MrgprX2 in mast cells drives degranulation and chemotaxis, contributing to the local release of proangiogenic factors [60]. These factors not only promote vascularization but also modulate local inflammatory responses, ensuring a balanced immune environment conducive to efficient wound healing (Figure 2). It is worth noting that in a mouse wound-healing model, AMP-IBP5 significantly accelerated wound closure with visible improvement observed as early as day 4 post-administration [11]. In contrast, hBD-3 required six days to achieve a similar level of tissue repair [32].

Notably, AMP-IBP5 has demonstrated therapeutic potential in the treatment of diabetic ulcers, which is one of the most severe and refractory complications, often resulting in chronically infected lesions that can lead to limb amputations. In diabetic mouse models, AMP-IBP5 significantly accelerated wound healing 8 days after injury and promoted angiogenesis, a crucial process that is often impaired in hyperglycemic environments [11]. The therapeutic effects of AMP-IBP5 in diabetic wound healing appear to involve multiple mechanisms that target the key pathological factors underlying impaired wound closure.

Hyperglycemia impairs neovascularization in diabetic wounds by suppressing key angiogenic factors, including angiogenin (ANG) and VEGF. AMP-IBP5 counteracts this angiogenic deficiency by upregulating ANG and VEGF expression, resulting in a marked increase in CD31-positive blood vessels within diabetic wound tissue [11]. This increased vascularization improves oxygen and nutrient delivery, creating a more favorable microenvironment for tissue repair. In diabetic wounds where the expression of AMPs, including the parent protein of AMP-IBP5, IGFBP-5, is decreased, the administration of AMP-IBP5 restores keratinocyte function by counteracting high-glucose-induced suppression. Through EGFR activation and MAPK pathway phosphorylation (ERK, JNK, and p38), AMP-IBP5 promotes keratinocyte migration and proliferation, accelerating re-epithelialization and wound closure [11]. Given its molecular mechanisms, particularly the promotion of VEGF and ANG expression, the effectiveness of AMP-IBP5 in diabetic models raises the possibility of broader applications to other metabolic or systemic disorders characterized by impaired wound healing. For example, conditions such as obesity, chronic kidney disease, or aging-associated tissue dysfunction are associated with similar deficits in angiogenesis and cellular regeneration. Collectively, investigating the potential of AMP-IBP5 across these contexts could uncover novel therapeutic strategies for managing wound-related complications beyond diabetes.

## 4. Role of AMP-IBP5 in Other Inflammatory Skin Diseases

AMP-IBP5 represents a critical molecular link between wound healing and inflammatory skin diseases because of its dual capacity to promote tissue regeneration and modulate inflammation. Wound healing and chronic skin conditions, such as atopic dermatitis (AD) and psoriasis, share overlapping pathological features, such as immune dysregulation, impaired barrier function, and aberrant cytokine production [61,62,63,64,65]. The ability of AMP-IBP5 to strengthen the skin barrier, reduce inflammation, and promote cellular regeneration makes it uniquely suited to address these shared challenges [66]. By addressing the underlying similarities in pathophysiology, AMP-IBP5 emerges as a promising therapeutic candidate that bridges wound management and inflammatory skin disease treatment.

### 4.1. AD

AD is a chronic inflammatory skin disorder characterized by a complex pathogenesis, including gene alterations, skin barrier dysfunction, skin microbiome abnormalities, T helper type 2 (Th2) inflammation, and neuroimmune interactions [67,68]. Microarray analysis reveals that the expression of IGFBP-5, the precursor of AMP-IBP5, is significantly downregulated in the lesional skin of patients with AD compared to nonlesional sites, indicating that AMP-IBP5 may be involved in AD pathogenesis. Further investigation has demonstrated that AMP-IBP5 restores skin barrier function by increasing the expression and distribution of tight junction (TJ)-related proteins such as claudin-1, occludin, and *zonula occludens* (ZO)-1 in both in vitro and in vivo AD models. This effect is mediated through the LRP1-dependent activation of atypical protein kinase Cζ and Ras-related C3 botulinum toxin substrate 1, leading to reduced skin permeability to allergens and irritants and limiting transepidermal water loss [66]. This targeted increase in TJ proteins underscores the therapeutic potential of AMP-IBP5 in directly addressing the barrier dysfunction associated with AD pathogenesis.

While many AMPs can exacerbate inflammation in skin diseases by activating immune cells, AMP-IBP5 displays an intriguing anti-inflammatory profile in the context of AD. Immune dysregulation in AD is primarily driven by Th2-skewed inflammation, characterized by the overproduction of key cytokines such as IL-4 and IL-13. Skin-derived AMPs such as hBD-2 and LL-37 have been shown to increase the expression of proinflammatory cytokines, including IL-4, IL-13, and IL-31 by T cells and mast cells, which are characteristic of AD pathology [36,69]. However, AMP-IBP5 appears to suppress the above inflammatory cascade. In a mouse model of AD induced by dinitrochlorobenzene (DNCB), AMP-IBP5 administration reduced the levels of IL-4, IL-13, and IL-33, all of which are involved in AD-related inflammation. Thymic stromal lymphopoietin (TSLP), a cytokine highly expressed in the epidermis of patients with AD and known to promote Th2 immune responses [70], was also significantly downregulated following AMP-IBP5 treatment in the lesional skin of AD mice. In addition, AMP-IBP5 decreased the infiltration of CD4^+^ T cells and mast cells in AD lesional skin and reduced total serum IgE levels, suggesting a comprehensive anti-inflammatory effect [66].

Itching is often described as the hallmark of AD. IL-31 and TSLP, in particular, are associated with AD-related itching [71], and their suppression by AMP-IBP5 resulted in reduced pruritus in mice with AD [66]. These effects highlight the multifunctionality of AMP-IBP5 in alleviating AD-like symptoms by enhancing skin barrier integrity, reducing inflammation, and relieving uncomfortable itching symptoms, thereby highlighting its potential as a promising therapeutic agent for AD management.

### 4.2. Psoriasis

Psoriasis is a long-lasting skin disease characterized by excessive proliferation of keratinocytes (epidermal hyperplasia), the formation of erythematous plaques, abnormal epidermal differentiation, and significant infiltration of immune cells, particularly by neutrophils [72]. In contrast to other AMPs, such as hBD-2, hBD-3, and LL-37, which are typically upregulated in psoriatic lesions [73,74,75], IGFBP-5 is notably downregulated in psoriatic skin, suggesting a distinct and potentially regulatory role in the pathogenesis of psoriasis. A recent study has shown that the subcutaneous administration of AMP-IBP5 in a psoriatic mouse model led to significant improvement in several hallmark features of the disease. These included the reduction of dry scales, erythematous plaques, epidermal thickness, blood vessel hyperplasia in the dermis, and neutrophil infiltration. Furthermore, AMP-IBP5 treatment was associated with the downregulation of key keratinocyte differentiation markers (involucrin and loricrin), pro-inflammatory cytokine (TNF-α), LL-37, and several angiogenesis factors in the psoriatic lesional skin [76]. These findings indicate that AMP-IBP5 may provide therapeutic effects in psoriasis not only by alleviating clinical symptoms but also by modulating the underlying inflammatory and proliferative pathways.

A key mechanism underlying the therapeutic effects of AMP-IBP5 involves the LRP1 signaling pathway. In psoriatic lesions, LRP1 expression is markedly decreased compared to normal or nonlesional skin, suggesting that its downregulation may contribute to persistent inflammation and disease progression. Notably, when mice were administered the receptor-associated protein (RAP), an antagonist of LRP1, the therapeutic benefits of AMP-IBP5 were abolished, and the psoriatic symptoms were exacerbated [76]. These findings provide compelling evidence that AMP-IBP5 shows its beneficial effects in psoriasis via LRP1-dependent mechanisms, highlighting LRP1 as a therapeutic target in the treatment of psoriasis.

## 5. Current Challenges and Possible Solutions

In addition to their well-known antimicrobial properties against various pathogens, AMPs exhibit numerous immunomodulatory functions, acting as both anti-inflammatory and proinflammatory agents, which makes them a double-edged sword in therapeutic applications [77]. Regarding AMP-mediated anti-inflammatory effects, for example, LL-37 is known to suppress the activity of proinflammatory cytokines, including IL-4, IL-12, TNF-α, and IFN-γ across various cell types [78,79]. Similarly, AMP-IBP5 effectively downregulates cytokine production, such as IL-4, IL-13, IL-31, IL-33, and TSLP, in a DNCB-induced AD mouse model [66].

On the other hand, the potential proinflammatory roles of AMPs cannot be overlooked. For instance, hBDs have been reported to stimulate the T-cell production of cytokines such as IL-4, IL-13, IL-22, and IL-31, all of which are implicated in AD pathogenesis [80]. LL-37 has similarly been associated with elevated levels of proinflammatory cytokines, including IL-1β, IL-12, and IL-18. In addition, LL-37 can trigger the degranulation of mast cells, leading to the release of histamine and other inflammatory mediators, thereby amplifying the inflammatory cascades [81]. Notably, IGFBP-5, the parent protein of AMP-IBP5, has been shown to exert proinflammatory effects in lung tissue [66]. However, the specific proinflammatory potential of AMP-IBP5 itself remains largely unexplored and warrants further investigation to clarify its immunomodulatory profile.

For clinical translation, establishing an effective and patient-friendly delivery method is essential for every functional biomaterial. In the case of AMP-IBP5, both subcutaneous injection and topical application have been investigated as potential administration routes. Interestingly, one study reported that there was no significant change in the improvement of dermatitis-like symptoms between subcutaneous injection and the topical administration of AMP-IBP5 [66], indicating that non-invasive methods may be equally effective. Although this peptide has a molecular weight of 2655 Daltons, which exceeds the conventional 500 Daltons threshold for transdermal absorption [82], it has demonstrated the ability to permeate the skin when formulated with acetic acid, a well-known skin penetration enhancer [83]. Furthermore, the compromised skin barrier commonly observed in AD may enhance its penetration through the skin. These observations underscore the promise of topical application as a practical and potentially patient-preferred method for AMP-IBP5 delivery. However, additional preclinical and clinical studies are necessary to fully elucidate its long-term therapeutic efficacy, optimal formulation strategies, and precise mechanisms in the context of AD.

AMPs are easily degraded by proteolytic enzymes in physiological environments, which significantly compromises their bioavailability and therapeutic efficacy [84]. This inherent instability necessitates the development of delivery systems or molecular modifications to enhance their stability and prolong their functional lifespan in vivo. Beyond nanoparticle encapsulation, other chemical strategies, such as PEGylation, which involves attaching polyethylene glycol chains to the peptide, have demonstrated efficacy in protecting AMPs from proteolytic degradation and extending their half-life [85]. Moreover, structural modifications such as peptide backbone cyclization or the incorporation of D-amino acids can confer resistance to enzymatic cleavage without compromising biological activity [86,87,88]. Another promising advancement is the development of hydrogel-based delivery systems, which provide controlled release of AMPs while maintaining their bioactivity [89,90]. Collectively, these approaches provide critical solutions to the stability challenges faced by AMPs and may facilitate the successful therapeutic application of AMP-IBP5.

## 6. Conclusions

AMPs have emerged as promising agents in wound management because of their dual roles in pathogen defense and cellular modulation within the wound-healing cascade. Among these AMPs, AMP-IBP5 is distinguished by superior antimicrobial potency and multifaceted biological functions, including increased keratinocyte and fibroblast activity, robust angiogenic effects, and immunomodulatory properties. In addition, AMP-IBP5 has demonstrated therapeutic potential in managing chronic inflammatory skin conditions such as AD and psoriasis. Through its interaction with the LRP1 pathway, AMP-IBP5 promotes skin barrier integrity by increasing the expression of TJ proteins such as claudin-1 and ZO-1, which fortify intercellular connections and reduce permeability to external irritants. Additionally, LRP1 activation by AMP-IBP5 modulates inflammatory signaling pathways, leading to a reduction in the levels of proinflammatory cytokines such as IL-6 and TNF-α. This dual action not only strengthens the structural defense of the skin but also creates a more balanced immune environment, addressing the core pathophysiological aspects of skin disorders. Continued research into AMP-IBP5 molecular mechanisms and their clinical efficacy could pave the way for the development of AMP-IBP5 as a next-generation therapeutic agent in dermatology, offering hope for improved patient care in complex and persistent skin conditions.

## Data Availability

No new data were created or analyzed in this study. Data sharing is not applicable to this article.

## References

[1] Mangoni M.L., McDermott A.M., Zasloff M. (2016). Antimicrobial Peptides and Wound Healing: Biological and Therapeutic Considerations. Exp. Dermatol..

[2] Reinke J.M., Sorg H. (2012). Wound Repair and Regeneration. Eur. Surg. Res..

[3] Rodrigues M., Kosaric N., Bonham C.A., Gurtner G.C. (2019). Wound Healing: A Cellular Perspective. Physiol. Rev..

[4] Soliman A.M., Barreda D.R. (2022). Acute Inflammation in Tissue Healing. Int. J. Mol. Sci..

[5] Solarte David V.A., Güiza-Argüello V.R., Arango-Rodríguez M.L., Sossa C.L., Becerra-Bayona S.M. (2022). Decellularized Tissues for Wound Healing: Towards Closing the Gap between Scaffold Design and Effective Extracellular Matrix Remodeling. Front. Bioeng. Biotechnol..

[6] Sen C.K. (2019). Human Wounds and Its Burden: An Updated Compendium of Estimates. Adv. Wound Care.

[7] Sen C.K., Gordillo G.M., Roy S., Kirsner R., Lambert L., Hunt T.K., Gottrup F., Gurtner G.C., Longaker M.T. (2009). Human Skin Wounds: A Major and Snowballing Threat to Public Health and the Economy. Wound Repair. Regen..

[8] Guest J.F., Fuller G.W., Vowden P. (2020). Cohort Study Evaluating the Burden of Wounds to the UK’s National Health Service in 2017/2018: Update from 2012/2013. BMJ Open.

[9] Frykberg R.G., Banks J. (2015). Challenges in the Treatment of Chronic Wounds. Adv. Wound Care.

[10] de Souza G.S., de Jesus Sonego L., Santos Mundim A.C., de Miranda Moraes J., Sales-Campos H., Lorenzón E.N. (2022). Antimicrobial-Wound Healing Peptides: Dual-Function Molecules for the Treatment of Skin Injuries. Peptides.

[11] Yue H., Song P., Sutthammikorn N., Umehara Y., Trujillo-Paez J.V., Nguyen H.L.T., Takahashi M., Peng G., Ikutama R., Okumura K. (2022). Antimicrobial Peptide Derived from Insulin-Like Growth Factor-Binding Protein 5 Improves Diabetic Wound Healing. Wound Repair. Regen..

[12] Osaki T., Sasaki K., Minamino N. (2011). Peptidomics-Based Discovery of an Antimicrobial Peptide Derived from Insulin-like Growth Factor-Binding Protein 5. J. Proteome Res..

[13] Zhang Q.Y., Yan Z.B., Meng Y.M., Hong X.Y., Shao G., Ma J.J., Cheng X.R., Liu J., Kang J., Fu C.Y. (2021). Antimicrobial Peptides: Mechanism of Action, Activity and Clinical Potential. Mil. Med. Res..

[14] Adp3, Antimicrobial Peptides Database. https://aps.unmc.edu/.

[15] Schauber J., Gallo R.L. (2008). Antimicrobial Peptides and the Skin Immune Defense System. J. Allergy Clin. Immunol..

[16] Yamasaki K., Gallo R.L. (2008). Antimicrobial Peptides in Human Skin Disease. Eur. J. Dermatol..

[17] Schittek B., Hipfel R., Sauer B., Bauer J., Kalbacher H., Stevanovic S., Schirle M., Schroeder K., Blin N., Meier F. (2001). Dermcidin: A Novel Human Antibiotic Peptide Secreted by Sweat Glands. Nat. Immunol..

[18] Schittek B., Paulmann M., Senyürek I., Steffen H. (2008). The Role of Antimicrobial Peptides in Human Skin and in Skin Infectious Diseases. Infect. Disord. Drug Targets.

[19] Vineeth Kumar T.V., Sanil G. (2017). A Review of the Mechanism of Action of Amphibian Antimicrobial Peptides Focusing on Peptide-Membrane Interaction and Membrane Curvature. Curr. Protein Pept. Sci..

[20] Qu H., Yao Q., Chen T., Wu H., Liu Y., Wang C., Dong A. (2024). Current Status of Development and Biomedical Applications of Peptide-Based Antimicrobial Hydrogels. Adv. Colloid. Interface Sci..

[21] Bin Hafeez A., Jiang X., Bergen P.J., Zhu Y. (2021). Antimicrobial Peptides: An Update on Classifications and Databases. Int. J. Mol. Sci..

[22] Li J., Hu S., Jian W., Xie C., Yang X. (2021). Plant Antimicrobial Peptides: Structures, Functions, and Applications. Bot. Stud..

[23] Rowe-Magnus D.A., Kao A.Y., Prieto A.C., Pu M., Kao C. (2019). Cathelicidin Peptides Restrict Bacterial Growth Via Membrane Perturbation and Induction of Reactive Oxygen Species. mBio.

[24] Sass V., Schneider T., Wilmes M., Körner C., Tossi A., Novikova N., Shamova O., Sahl H.G. (2010). Human Beta-Defensin 3 Inhibits Cell Wall Biosynthesis in Staphylococci. Infect. Immun..

[25] Zielke C., Nielsen J.E., Lin J.S., Barron A.E. (2024). Between Good and Evil: Complexation of the Human Cathelicidin LL-37 with Nucleic Acids. Biophys. J..

[26] Komatsu T., Salih E., Helmerhorst E.J., Offner G.D., Oppenheim F.G. (2011). Influence of Histatin 5 on Candida Albicans Mitochondrial Protein Expression Assessed by Quantitative Mass Spectrometry. J. Proteome Res..

[27] Zhang L.-J., Gallo R.L. (2016). Antimicrobial Peptides. Curr. Biol..

[28] Fjell C.D., Hiss J.A., Hancock R.E., Schneider G. (2011). Designing Antimicrobial Peptides: Form Follows Function. Nat. Rev. Drug Discov..

[29] Niyonsaba F., Ushio H., Nakano N., Ng W., Sayama K., Hashimoto K., Nagaoka I., Okumura K., Ogawa H. (2007). Antimicrobial Peptides Human Beta-Defensins Stimulate Epidermal Keratinocyte Migration, Proliferation and Production of Proinflammatory Cytokines and Chemokines. J. Investig. Dermatol..

[30] Aung G., Niyonsaba F., Ushio H., Kajiwara N., Saito H., Ikeda S., Ogawa H., Okumura K. (2011). Catestatin, a Neuroendocrine Antimicrobial Peptide, Induces Human Mast Cell Migration, Degranulation and Production of Cytokines and Chemokines. Immunology.

[31] Tokumaru S., Sayama K., Shirakata Y., Komatsuzawa H., Ouhara K., Hanakawa Y., Yahata Y., Dai X., Tohyama M., Nagai H. (2005). Induction of Keratinocyte Migration Via Transactivation of the Epidermal Growth Factor Receptor by the Antimicrobial Peptide LL-37. J. Immunol..

[32] Takahashi M., Umehara Y., Yue H., Trujillo-Paez J.V., Peng G., Nguyen H.L.T., Ikutama R., Okumura K., Ogawa H., Ikeda S. (2021). The Antimicrobial Peptide Human β-Defensin-3 Accelerates Wound Healing by Promoting Angiogenesis, Cell Migration, and Proliferation through the FGFR/JAK2/STAT3 Signaling Pathway. Front. Immunol..

[33] Hu Q., Chen C., Lin Z., Zhang L., Guan S., Zhuang X., Dong G., Shen J. (2023). The Antimicrobial Peptide Esculentin-1a(1-21)NH_2_ Stimulates Wound Healing by Promoting Angiogenesis through the PI3K/AKT Pathway. Biol. Pharm. Bull..

[34] Choi K.Y., Mookherjee N. (2012). Multiple Immune-Modulatory Functions of Cathelicidin Host Defense Peptides. Front. Immunol..

[35] Scott M.G., Davidson D.J., Gold M.R., Bowdish D., Hancock R.E. (2002). The Human Antimicrobial Peptide LL-37 Is a Multifunctional Modulator of Innate Immune Responses. J. Immunol..

[36] Niyonsaba F., Ushio H., Hara M., Yokoi H., Tominaga M., Takamori K., Kajiwara N., Saito H., Nagaoka I., Ogawa H. (2010). Antimicrobial Peptides Human Beta-Defensins and Cathelicidin LL-37 Induce the Secretion of a Pruritogenic Cytokine IL-31 by Human Mast Cells. J. Immunol..

[37] Bayer A., Lammel J., Tohidnezhad M., Lippross S., Behrendt P., Klüter T., Pufe T., Cremer J., Jahr H., Rademacher F. (2017). The Antimicrobial Peptide Human Beta-Defensin-3 Is Induced by Platelet-Released Growth Factors in Primary Keratinocytes. Mediators Inflamm..

[38] Carretero M., Escámez M.J., García M., Duarte B., Holguín A., Retamosa L., Jorcano J.L., Río M.D., Larcher F. (2008). In Vitro and in Vivo Wound Healing-Promoting Activities of Human Cathelicidin LL-37. J. Investig. Dermatol..

[39] Ramos R., Silva J.P., Rodrigues A.C., Costa R., Guardão L., Schmitt F., Soares R., Vilanova M., Domingues L., Gama M. (2011). Wound Healing Activity of the Human Antimicrobial Peptide LL37. Peptides.

[40] Berthet M., Gauthier Y., Lacroix C., Verrier B., Monge C. (2017). Nanoparticle-Based Dressing: The Future of Wound Treatment?. Trends Biotechnol..

[41] Sánchez-Peña F.J., Romero-Tlalolini M.L.Á., Torres-Aguilar H., Cruz-Hernández D.S., Baltiérrez-Hoyos R., Sánchez-Aparicio S.R., Aquino-Domínguez A.S., Aguilar-Ruiz S.R. (2023). LL-37 Triggers Antimicrobial Activity in Human Platelets. Int. J. Mol. Sci..

[42] Salamah M.F., Ravishankar D., Kodji X., Moraes L.A., Williams H.F., Vallance T.M., Albadawi D.A., Vaiyapuri R., Watson K., Gibbins J.M. (2018). The Endogenous Antimicrobial Cathelicidin LL37 Induces Platelet Activation and Augments Thrombus Formation. Blood Adv..

[43] Pavlovic V., Ciric M., Jovanovic V., Stojanovic P. (2016). Platelet Rich Plasma: A Short Overview of Certain Bioactive Components. Open Med..

[44] Kraemer B.F., Campbell R.A., Schwertz H., Cody M.J., Franks Z., Tolley N.D., Kahr W.H., Lindemann S., Seizer P., Yost C.C. (2011). Novel Anti-Bacterial Activities of β-Defensin 1 in Human Platelets: Suppression of Pathogen Growth and Signaling of Neutrophil Extracellular Trap Formation. PLoS Pathog..

[45] de Oliveira S., Rosowski E.E., Huttenlocher A. (2016). Neutrophil Migration in Infection and Wound Repair: Going Forward in Reverse. Nat. Rev. Immunol..

[46] Koh T.J., DiPietro L.A. (2011). Inflammation and Wound Healing: The Role of the Macrophage. Expert. Rev. Mol. Med..

[47] Roupé K.M., Nybo M., Sjöbring U., Alberius P., Schmidtchen A., Sørensen O.E. (2010). Injury Is a Major Inducer of Epidermal Innate Immune Responses during Wound Healing. J. Investig. Dermatol..

[48] Wang Y., Graves D.T. (2020). Keratinocyte Function in Normal and Diabetic Wounds and Modulation by FOXO1. J. Diabetes Res..

[49] Werner S., Krieg T., Smola H. (2007). Keratinocyte-Fibroblast Interactions in Wound Healing. J. Investig. Dermatol..

[50] Kao H.K., Chen B., Murphy G.F., Li Q., Orgill D.P., Guo L. (2011). Peripheral Blood Fibrocytes: Enhancement of Wound Healing by Cell Proliferation, Re-Epithelialization, Contraction, and Angiogenesis. Ann. Surg..

[51] Shams F., Moravvej H., Hosseinzadeh S., Mostafavi E., Bayat H., Kazemi B., Bandehpour M., Rostami E., Rahimpour A., Moosavian H. (2022). Overexpression of VEGF in Dermal Fibroblast Cells Accelerates the Angiogenesis and Wound Healing Function: In Vitro and in Vivo Studies. Sci. Rep..

[52] Tomioka H., Nakagami H., Tenma A., Saito Y., Kaga T., Kanamori T., Tamura N., Tomono K., Kaneda Y., Morishita R. (2014). Novel Anti-Microbial Peptide SR-0379 Accelerates Wound Healing Via the PI3 kinase/Akt/mTOR Pathway. PLoS ONE.

[53] Ding H., Wu T. (2018). Insulin-Like Growth Factor Binding Proteins in Autoimmune Diseases. Front. Endocrinol..

[54] IGFBP5, the Human Protein Atlas. https://www.proteinatlas.org/ENSG00000115461-IGFBP5.

[55] Lin S.C., Wang C.P., Chen Y.M., Lu S.Y., Fann M.J., Liu C.J., Kao S.Y., Chang K.W. (2002). Regulation of IGFBP-5 Expression during Tumourigenesis and Differentiation of Oral Keratinocytes. J. Pathol..

[56] Dos Santos Cabrera M.P., Arcisio-Miranda M., Broggio Costa S.T., Konno K., Ruggiero J.R., Procopio J., Ruggiero Neto J. (2008). Study of the Mechanism of Action of Anoplin, a Helical Antimicrobial Decapeptide with Ion Channel-Like Activity, and the Role of the Amidated C-Terminus. J. Pept. Sci..

[57] Bauer F., Schweimer K., Klüver E., Conejo-Garcia J.R., Forssmann W.G., Rösch P., Adermann K., Sticht H. (2001). Structure Determination of Human and Murine Beta-Defensins Reveals Structural Conservation in the Absence of Significant Sequence Similarity. Protein Sci..

[58] Chieosilapatham P., Niyonsaba F., Kiatsurayanon C., Okumura K., Ikeda S., Ogawa H. (2017). The Antimicrobial Peptide Derived from Insulin-Like Growth Factor-Binding Protein 5, AMP-IBP5, Regulates Keratinocyte Functions through Mas-Related Gene X Receptors. J. Dermatol. Sci..

[59] Chieosilapatham P., Yue H., Ikeda S., Ogawa H., Niyonsaba F. (2020). Involvement of the Lipoprotein Receptor Lrp1 in AMP-IBP5-Mediated Migration and Proliferation of Human Keratinocytes and Fibroblasts. J. Dermatol. Sci..

[60] Niyonsaba F., Song P., Yue H., Sutthammikorn N., Umehara Y., Okumura K., Ogawa H. (2020). Antimicrobial Peptide Derived from Insulin-Like Growth Factor-Binding Protein 5 Activates Mast Cells Via Mas-Related G Protein-Coupled Receptor X2. Allergy.

[61] Raziyeva K., Kim Y., Zharkinbekov Z., Kassymbek K., Jimi S., Saparov A. (2021). Immunology of Acute and Chronic Wound Healing. Biomolecules.

[62] Larouche J., Sheoran S., Maruyama K., Martino M.M. (2018). Immune Regulation of Skin Wound Healing: Mechanisms and Novel Therapeutic Targets. Adv. Wound Care.

[63] Tsai Y.C., Tsai T.F. (2022). Overlapping Features of Psoriasis and Atopic Dermatitis: From Genetics to Immunopathogenesis to Phenotypes. Int. J. Mol. Sci..

[64] Barrientos S., Stojadinovic O., Golinko M.S., Brem H., Tomic-Canic M. (2008). Growth Factors and Cytokines in Wound Healing. Wound Repair. Regen..

[65] Nirenjen S., Narayanan J., Tamilanban T., Subramaniyan V., Chitra V., Fuloria N.K., Wong L.S., Ramachawolran G., Sekar M., Gupta G. (2023). Exploring the Contribution of Pro-Inflammatory Cytokines to Impaired Wound Healing in Diabetes. Front. Immunol..

[66] Nguyen H.L.T., Peng G., Trujillo-Paez J.V., Yue H., Ikutama R., Takahashi M., Umehara Y., Okumura K., Ogawa H., Ikeda S. (2023). The Antimicrobial Peptide AMP-IBP5 Suppresses Dermatitis-Like Lesions in a Mouse Model of Atopic Dermatitis through the Low-Density Lipoprotein Receptor-Related Protein-1 Receptor. Int. J. Mol. Sci..

[67] Ständer S. (2021). Atopic Dermatitis. N. Engl. J. Med..

[68] Kim J., Kim B.E., Leung D.Y.M. (2019). Pathophysiology of Atopic Dermatitis: Clinical Implications. Allergy Asthma Proc..

[69] Kanda N., Hau C.S., Tada Y., Sato S., Watanabe S. (2012). Decreased Serum LL-37 and Vitamin D3 Levels in Atopic Dermatitis: Relationship between Il-31 and Oncostatin M. Allergy.

[70] Luo J., Zhu Z., Zhai Y., Zeng J., Li L., Wang D., Deng F., Chang B., Zhou J., Sun L. (2023). The Role of TSLP in Atopic Dermatitis: From Pathogenetic Molecule to Therapeutical Target. Mediators Inflamm..

[71] Hashimoto T., Yokozeki H., Karasuyama H., Satoh T. (2023). IL-31-Generating Network in Atopic Dermatitis Comprising Macrophages, Basophils, Thymic Stromal Lymphopoietin, and Periostin. J. Allergy Clin. Immunol..

[72] Boehncke W.H., Schön M.P. (2015). Psoriasis. Lancet.

[73] Hollox E.J., Huffmeier U., Zeeuwen P.L., Palla R., Lascorz J., Rodijk-Olthuis D., van de Kerkhof P.C., Traupe H., de Jongh G., den Heijer M. (2008). Psoriasis Is Associated with Increased Beta-Defensin Genomic Copy Number. Nat. Genet..

[74] Fuentes-Duculan J., Bonifacio K.M., Hawkes J.E., Kunjravia N., Cueto I., Li X., Gonzalez J., Garcet S., Krueger J.G. (2017). Autoantigens ADAMTSL5 and LL37 Are Significantly Upregulated in Active Psoriasis and Localized with Keratinocytes, Dendritic Cells and Other Leukocytes. Exp. Dermatol..

[75] Gläser R., Meyer-Hoffert U., Harder J., Cordes J., Wittersheim M., Kobliakova J., Fölster-Holst R., Proksch E., Schröder J.M., Schwarz T. (2009). The Antimicrobial Protein Psoriasin (S100A7) Is Upregulated in Atopic Dermatitis and after Experimental Skin Barrier Disruption. J. Investig. Dermatol..

[76] Yoshiba S., Peng G.E., Niyonsaba F. (2023). A Skin-Derived Antimicrobial Peptide Derived from Insulin-Like Growth Factor-Binding Protein 5 (AMP-IBP5) as Therapeutic Candidate for Psoriasis. Juntendo Iji Zasshi.

[77] Liang W., Diana J. (2020). The Dual Role of Antimicrobial Peptides in Autoimmunity. Front. Immunol..

[78] Kahlenberg J.M., Kaplan M.J. (2013). Little Peptide, Big Effects: The Role of LL-37 in Inflammation and Autoimmune Disease. J. Immunol..

[79] Nagaoka I., Tamura H., Reich J. (2020). Therapeutic Potential of Cathelicidin Peptide LL-37, an Antimicrobial Agent, in a Murine Sepsis Model. Int. J. Mol. Sci..

[80] Chieosilapatham P., Ogawa H., Niyonsaba F. (2017). Current Insights into the Role of Human β-Defensins in Atopic Dermatitis. Clin. Exp. Immunol..

[81] Niyonsaba F., Kiatsurayanon C., Chieosilapatham P., Ogawa H. (2017). Friends or Foes? Host Defense (Antimicrobial) Peptides and Proteins in Human Skin Diseases. Exp. Dermatol..

[82] Bos J.D., Meinardi M.M. (2000). The 500 Dalton Rule for the Skin Penetration of Chemical Compounds and Drugs. Exp. Dermatol..

[83] Wang M.Y., Yang Y.Y., Heng P.W. (2004). Role of Solvent in Interactions between Fatty Acids-Based Formulations and Lipids in Porcine Stratum Corneum. J. Control Release.

[84] Canè C., Tammaro L., Duilio A., Di Somma A. (2024). Investigation of the Mechanism of Action of AMPs from Amphibians to Identify Bacterial Protein Targets for Therapeutic Applications. Antibiotics.

[85] Manteghi R., Pallagi E., Olajos G., Csóka I. (2020). Pegylation and Formulation Strategy of Anti-Microbial Peptide (AMP) According to the Quality by Design Approach. Eur. J. Pharm. Sci..

[86] Melchionna M., Styan K.E., Marchesan S. (2016). The Unexpected Advantages of Using D-Amino Acids for Peptide Self- Assembly into Nanostructured Hydrogels for Medicine. Curr. Top. Med. Chem..

[87] Li Y., Liu T., Liu Y., Tan Z., Ju Y., Yang Y., Dong W. (2019). Antimicrobial Activity, Membrane Interaction and Stability of the D-Amino Acid Substituted Analogs of Antimicrobial Peptide W3R6. J. Photochem. Photobiol. B.

[88] Kremsmayr T., Aljnabi A., Blanco-Canosa J.B., Tran H.N.T., Emidio N.B., Muttenthaler M. (2022). On the Utility of Chemical Strategies to Improve Peptide Gut Stability. J. Med. Chem..

[89] Lin X., Zhang X., Wang Y., Chen W., Zhu Z., Wang S. (2025). Hydrogels and Hydrogel-Based Drug Delivery Systems for Promoting Refractory Wound Healing: Applications and Prospects. Int. J. Biol. Macromol..

[90] Wu L., He Y., Mao H., Gu Z. (2022). Bioactive Hydrogels Based on Polysaccharides and Peptides for Soft Tissue Wound Management. J. Mater. Chem. B.

